# Lipidomic profiling reveals phenotypic diversity and nutritional benefits in *Ficus carica* L. (Fig.) seed cultivars

**DOI:** 10.3389/fpls.2023.1229994

**Published:** 2023-11-10

**Authors:** Ahmed Irchad, Rachida Ouaabou, Rachid Aboutayeb, Rachid Razouk, Karim Houmanat, Lahcen Hssaini

**Affiliations:** ^1^Faculty of Sciences and Techniques, University of Comoros, Moroni, Comoros; ^2^Hygiene and Food Safety Department, National Research Institute for Agriculture, Fisheries and Environment (INRAPE), Ex CEFADER, M’dé, Ngazidja, Moroni, Comoros; ^3^Environmental Technologies, Biotechnology and Valorization of Bio-Resources Team, Faculty of Sciences and Techniques Al-Hoceïma, Abdelmalek Essâadi University, Al-Hoceïma, Morocco; ^4^Agro-Food Technology and Quality Laboratory, Regional Center of Agricultural Research of Meknes, National Institute of Agricultural Research, Rabat, Morocco

**Keywords:** chemometrics, *Ficus carica* L, ionomic analysis, nutritional quality, seeds, vibrational spectroscopy

## Abstract

**Introduction:**

*Ficus carica* L. seeds are a substantial source of minor oil with high unsaturation levels and potent antioxidant properties. The study aims to evaluate the mineral composition, lipodomic profile, and vibrational fingerprints of 22 fig genotypes utilizing FTIR-ATR techniques and chemometrics.

**Methods:**

FTIR-ATR spectroscopy and chemometric techniques were employed to examine the phenotypic diversity of fig seeds. The investigation was performed in detail. The research analyzed twenty-two fig genotypes to assess their nutritional properties, genetic relationships, and potential applications.

**Results:**

The results demonstrate substantial nutritional benefits related to fig seeds, which could serve as genetic resources for selection programs for extracting vegetable oil and functional ingredients. Additionally, a detailed lipodomic profile analysis led to the categorization of the genotypes into four unique clusters. The study uncovered new insights regarding the nutritional composition of the samples, while also highlighting significant similarities and differences. The findings showcased the phenotypic diversity within the studied fig germplasm, which is likely attributed to underlying genetic factors. These accessions offer a valuable gene pool for future breeding programs and diverse applications involving fig seeds.

**Discussion:**

This work contributes to the selection of potential genotypes for scientific and industrial purposes. Furthermore, the application of FTIR and chemometrics revealed a noteworthy diversity of patterns, emphasizing the previously underestimated significance of this aspect in evaluating the chemodiversity of the species.

## Highlights

- Phenotypic diversity of seeds and fig seed oils were described for the first time.
- This first exploratory study screened 22 cultivars of fig seeds based on their proteins and mineral content and lipid properties using a combination of FTIR-ATR spectroscopic analysis and multivariate analysis.
- Lipochemical-based fingerprinting of fig seeds revealed the phenotypic diversity within the germplasm of the fig tree.
- The findings revealed the substantial nutritional benefits of fig seeds as a nutritious source of lipids, minerals and proteins, and their potentialities in genetic material selection programs.
- Results introduce FTIR-ATR spectroscopy combined with multivariate analysis as a convenient highly sensitive method and effective in fig seed cultivars discrimination and classification.

## 1 Introduction

Figs (*Ficus carica* L.) are commonly consumed fruits which are known for their sweet and flavorful taste, but the seeds contained within the fruits often go unnoticed (Hssaini et al., 2021a). These seeds, which vary in number and size, play a crucial role in determining the flavor and taste of figs, as well as their nutritional composition. These aspects are influenced by several factors, including the genotype of the fig, its ripening stage, and the mutualistic relationship between the fig and its pollinator wasp, which is known as caprification (Solomon et al., 2006; Gaaliche et al., 2011; Rosianski et al., 2016; Hssaini et al., 2022). Despite their contribution to the nutritional composition of figs, there have been very few studies on fig seeds (Hssaini et al., 2020; Hssaini et al., 2021a). Previous research has shown that fig seed oil is generally pale yellow in color and yields can vary significantly based on the genotype, with some genotypes yielding up to 30% oil content (Hssaini et al., 2020). The macro-qualitative components of fig seeds, including oil yield and lipochemical composition, are influenced by both genetic differences between seeds and geographical variations (Raihana et al., 2015; Górnaś and Rudzińska, 2016) as well as the pollination system (Hssaini et al., 2022). Compared to other fruit seeds, fig seeds have an oil yield that is similar to pumpkin (27.83%) (Alfawaz, 2004), honeydew (25%) (Yanty et al., 2008), and mangosteen (21.18%) (Ajayi et al., 2007), and higher than those of durian seeds (1.8%) (Berry, 1980), Opuntia ficus indica seeds (5.4 to 9.9%) (Taoufik et al., 2015), and guava (16%) (Prasad and Azeemoddin, 1994). With such high oil yields, fig seeds have the potential to be used for industrial purposes, much like other fruit seeds. Despite this, fig seeds have been relatively unstudied, and many questions remain unanswered, particularly regarding the large-scale impact of genotype on the nutritional components and oil yield of fig seeds.

The mineral composition of fig (*Ficus carica* L.) seeds has received limited attention, and there is a notable gap in the literature when it comes to investigating seeds from a wide range of genotypes. While several studies have estimated the concentration of mineral elements in different parts of the *Ficus carica* L. plant, including its leaves, aerial roots, and bark (Khan, 2011), the majority of research has predominantly focused on the entire fruit, particularly dried figs (Aljane and Ferchichi, 2007; Aljane and Ferchichi, 2009; Khan, 2011; Sadia et al., 2014; Lo Turco et al., 2020). To date, there have been very few studies dedicated to exploring the mineral composition of fig seeds, despite their importance in providing essential nutrients that cannot be synthesized by the human body and must be obtained through dietary sources or supplements to meet daily nutritional requirements (Chongtham et al., 2020). Scientific evidence suggests that a diet rich in minerals can play a crucial role in preventing and treating various diseases, such as diabetes, heart disease, stroke, and cancer (Tolonen, 1990; Branco et al., 2016). Macro-elements, including calcium, phosphorus, sodium, magnesium, and potassium, are involved in vital cellular transmission and signaling processes. Additionally, micro-elements, such as copper, iron, manganese, selenium, and zinc, serve as essential structural components of numerous enzymes (Gharibzahedi and Jafari, 2017). Given the potential health implications and the significance of mineral elements in the human diet, the scarcity of studies investigating the mineral composition of fig seeds is noteworthy. As such, the present study aims to address this gap in the literature by comprehensively evaluating the mineral content of Ficus carica L. seeds from various fig genotypes. By conducting such a study, we aim to shed light on the nutritional importance and potential health benefits associated with the consumption of fig seeds. The findings from this research may provide valuable insights into the unique mineral profiles of different fig genotypes, thus contributing to a deeper understanding of the overall nutritional composition of the fig tree and its potential applications in promoting human health.

Recently, phenotypic diversity of seeds and fig seed oils has been reported for only four genotypes (Hssaini et al., 2020; Hssaini et al., 2021a). This study represents the first exploratory work that screens 22 genotypes of fig seeds based on their proteins and mineral content and lipid properties using a combination of FTIR-ATR spectroscopic analysis and multivariate analysis. This study is crucial in providing information that could be useful in genetic improvement and identifying desirable traits for this atypical oilseed source. By conducting the genotypes under similar experimental conditions, our study overcomes the problem of environmental variability and captures the chemodiversity potentially linked to genetic factors. Using highly sensitive vibrational methods, such as FTIR-ATR spectroscopy, has been already effective in fig genotypes discrimination and classification (Hssaini et al., 2021a; Hssaini et al., 2021b; Hssaini et al., 2022). This novel study will contribute to the improvement and expansion of the scientific knowledge of fig seeds and will be of utmost importance since the chemical composition of fig seeds has received very little attention and no previous study has screened such a wide range of genotypes.

## 2 Materials and methods

### 2.1 Plant material and experimental design

Plant material used in this study involves 22 genotypes of fig trees from an ex-situ collection located in the Sais plain in Morocco and managed by the National Institute for Agricultural Research (INRA Morocco). The collection is 14 years old, trained to a cup shape, and planted in a randomized complete block design with three trees per genotype and a spacing of 5x3 meters. The collection comprised a diverse range of both exotic and locally sourced genotypes, as detailed in **Table 1**.

**Table 1:** List of exotic and local genotypes of fig (*Ficus carica* L.) trees used in this study.

Exotic genotypes	Local genotypes
Breval Blanca 2736	Ahra 2870
Grosse Dame Blanche 2953	Aicha Moussa 2208
Melissosyki 3074	EL Qoti Lezreq 2883
White Adriatic 102	Embar El Khal 2247
Lerida 2280	Hafer Jmel 2253
Bourjassate Noire	Nabout 2893
Rhoult 2216	INRA 2105
Adroulaniki 3073	INRA 2603
Kamalata 3075	INRA 2802
Bourqui 2210	
Hayoul 2265	
Amtalaa Arch 2210	
Breba Blanca	

### 2.2 Fruit sampling method

Fig fruits were harvested at their full ripening stage in the summer. The genotypes were carefully selected based on the quantity and size of seeds in their fruits. To determine full ripeness, the figs were evaluated for a reddish-purple coloration that covered three-quarters of the receptacle and ensured that the fruit could be easily separated from the twig. The fruits were randomly picked from various positions around the canopy at a height of 160 cm, ensuring that the sample was representative of the entire tree.

### 2.3 Extraction of seeds and oil

Manually peeling figs, the seeds were separated from the pulp through a technical ethanol solution (10%). The mixture was stirred for about 10 minutes, then allowed to settle until the seeds separated and floated to the surface. The yellow, round-shaped seeds were collected, thoroughly washed with distilled water, and left to dry at room temperature for 24 hours. For each sample, the extracted seeds were ground into a fine powder using an IKA A11 Basic type grinder. The fig seed oil was then extracted chemically using a Soxhlet apparatus. To extract the oil, 20g of the powder was mixed with 150ml of 99% pure n-hexane using cellulose cartridges. The extraction process took up to 4 hours, after which the solvent was evaporated using a Buchi rotavapor R-200 at 40°C. The oil weight was determined through the relationship provided by Chougui et al. (2013):


$$\text{Oil yield}\left(\%\right)=[({\text{M}}_{1}-{\text{M}}_{0})/{\text{M}}_{2}]*100$$

Where M_0_ refers to empty flask weight (g), M_1_ is the flask weight after solvent evaporation (g), and M_2_ is the seed powder weight (g). At the same time, the average oilcake weight was determined, and extracted oils were stored in dark glass bottles at 4°C until analysis.

### 2.4 Ionomic analysis

The Total Nitrogen (NT) content was quantified using the Kjeldahl method (Buchi, Switzerland). The Nitrate content was measured by complexing with chromotropic acid and analyzing the absorbance in a spectrophotometer (Spectronics, USA) set at 410 nm (Hadjidemetriou, 1982). The protein content was calculated based on the Nitrogen content, using the formula P = 6.25 * NT. The Phosphorus (P) content was determined using the yellow color method with NaOH to neutralize excessive acidity, measured using a spectrophotometer (Shimadzu, China) (Gupta et al., 1993). Other elements (K, Na, Ca, Mg, Fe, Cu, Zn, and Mn) were analyzed through a dry incineration method carried out at 50°C for 48 hours in an oven. The macroelements (K, Na, Ca, and Mg) were evaluated using a flame photometer (Model CL 378, Elico, India) after extraction with nitric acid from 250mg of dry samples (Knudsen et al., 1982). The microelements (Fe, Cu, Zn, and Mn) were evaluated by atomic absorption spectrometry (AAS) (Perkin Elmer Analyst 300, USA) (Estefan et al., 2013). Three measurements were taken for each sample to ensure the reliability of the analysis.

### 2.5 Spectral analyzes

#### 2.5.1 Mid-infrared spectroscopic measurements

The Fourier Transform Infrared (FTIR) spectra of the seeds were collected using a Bruker FTIR spectrometer Vertex 70 equipped with a diamond ATR attachment (Bruker Optics Inc., Ettlingen, Germany). The spectra were taken in the wavenumber range of 4000 to 450 cm^-1^, with a spectral resolution of 4 cm^-1^. At room temperature, 100mg of each sample was analyzed and the resulting spectrum was the mean of multiple replicates, each of which was an accumulation of 128 scans. The germanium crystal was in contact with the samples after applying the standard pressure setting. Before the sample evaluation, a background spectrum was collected and matched with the infrared spectrum of the empty germanium crystal surface, which was then automatically subtracted from the sample spectrum. The crystal cell was cleaned between spectral collections using technical ethanol and hot water, and then carefully dried with paper towels.

#### 2.5.2 Spectra processing

Prior to data analysis, the Standard Normal Variable (SNV) and Multiplicative Scattering Correction (MSC) procedure, a basic correction method, were performed to correct multiplicative interferences (Shen et al., 2018). An Attenuated Total Reflectance (ATR) correction procedure was applied using the following processing parameters: angle of incidence = 45°, ATR reflection number = 1, average refractive index of the sample = 1.5, maximum interaction = 50, and 1.8mm for the crystal surface. The important feature of ATR is the evanescent field, which occurs when infrared light reflects at the interface between a high refractive index material (ATR crystal) and a low refractive index material (sample) (De Nardo et al., 2009). The peaks in each spectrum, which indicate the presence of certain vibrational regions corresponding to the functional groups of the sample, including the integrated areas, were calculated using Essential FTIR software (version 3.50.183). The corrected spectra and peaks absorbance were plotted using OriginLab Pro v2019 software (OriginLab Corporation Inc.).

### 2.6 Statistical processing and chemometrics

To validate the results of our chemometric analysis, we first checked the normality and homogeneity of variance in our data. Next, we used IBM SPSS Statistics v22 software to conduct a one-way ANOVA to determine significant differences (P<0.05) between fig seed samples. The ionomic and FTIR data were then subjected to a principal component analysis (PCA) to uncover variables and spectral signatures that best explain the phenotypic diversity between samples. The PCA was performed using OriginLab Pro v2019 and 2D scatterplots were created to illustrate the classification patterns based on genetic seed variability. The PCA was significant because it reduced the dimensionality of the data set and allowed us to identify the signatures that reflect shared variance within the data set. Pearson’s correlation coefficient (r) was used to evaluate the associations between traits. Lastly, we performed a hierarchical cluster analysis (HCA) using Ward’s method based on Euclidean distance to cluster fig seed genotypes based on mineral composition and protein content. The results were displayed using a tree diagram (dendrogram) after standardizing the data.

## 3 Results and discussion

### 3.1 Seed yield per fruit and oil yield

Fig seeds are typically round and yellow in appearance. According to Hssaini et al. (2020), the average weight of a thousand seeds is around 1.14 ± 0.01g. However, as shown in **Table 2**, the weight of seeds per fig fruit can vary significantly based on the genotype. There were differences ranging from a minimum of 1.14 g/fruit for the “Grosse Dame Blanche” genotype to a maximum of 2.45 g/fruit for the “El Qoti Lezreq” genotype. On the other hand, few studies have evaluated the oil yield from fig seeds. Yarosh and Umarov (1971) found that the extraction produced 29.4% oil, while seeds from various regions in Turkey had oil yields ranging from 23.06 to 23.67% for the “Sarilop” cultivar (Nakilcioğlu-Tas, 2019). Joseph and Raj (2011) reported that the dried fig seeds had a fixed oil content of 30%. Hssaini et al. (2020) found that the oil content of four different fig seed cultivars from Morocco ranged from 21.54 to 29.65% and was characterized by a high content of linolenic acid. This high yield compared to the oil from other fruits makes fig seed oil a valuable component of the human diet and its commercial production should be encouraged due to its health benefits (Hssaini et al., 2020; Hssaini et al., 2021a). Despite these benefits, there are limited studies on fig seeds and the impact of cultivar on oil yield has not received much attention. However, our results are in line with previous studies. As seen in **Table 2**, the oil yield from fig seeds can vary greatly based on the cultivar, with the highest yields recorded by the “Bourqui” (39.68%) and “Bourjassate Noire” (38.44%) genotypes, while the genotypes “INRA 2105” and “INRA 2802” had lower yields of 15.06 and 16.66% respectively. These results suggest that the variability in yield can be attributed to the genetic diversity of the cultivars. Further research is necessary to fully understand the impact of cultivar diversity on the chemical composition and other qualities of this oil.

**Table 2:** Fig (*Ficus carica* L.) seeds yield (g/fruit) and oil content (%) of studied genotypes (mean values of three repetitions).

Genotypes	Seeds yield (g/fruit)	Oil yield (%)
Bourqui 2210	1,39 ±0,54	39,68 ±3,3
Melissosyki 3074	1,97 ±0,48	23,88 ±0,5
Embar El Khal 2247	2,09 ±0,75	34,13 ±0,4
Kamalata 3075	2,09 ±0,14	30,14 ±5,2
Adroulaniki 3073	1,94 ±0,48	28,72 ±4,5
Amtalaa Arch 2210	2,08 ±0,12	35,18 ±2,5
Lerida 2280	2,24 ±0,38	32,44 ±4,3
Breba Blanca	2,29 ±0,18	25,99 ±4,9
Hayoul 2265	2,17 ±0,87	26,55 ±4,2
INRA 2802	1,79 ±0,8	16,66 ±1,4
Bourjassate Noire	1,99 ±0,51	38,44 ±0,1
Breval Blanca 2736	2,04 ±0,3	35,28 ±5,3
Aicha Moussa 2208	1,39 ±0,89	34,2 ±3,6
Nabout 2893	2,44 ±0,6	32,58 ±4,2
Grosse Dame Blanche 2953	1,14 ±0,35	29,75 ±4,6
EL Qoti Lezreq 2883	2,45 ±0,02	28,19 ±2,6
Hafer Jmel 2253	2,23 ±0,4	30,39 ±1
Rhoult 2216	2,07 ±0,78	32,06 ±2,4
White Adriatic 102	2,27 ±0,93	23,05 ±3,3
INRA 2603	1,98 ±0,15	25,29 ±3,1
INRA 2105	1,96 ±0,09	15,06 ±2,1
Ahra 2870	2,24 ±0,05	34,93 ±5,2

### 3.2 Proteins and mineral content

**Figure 1:**
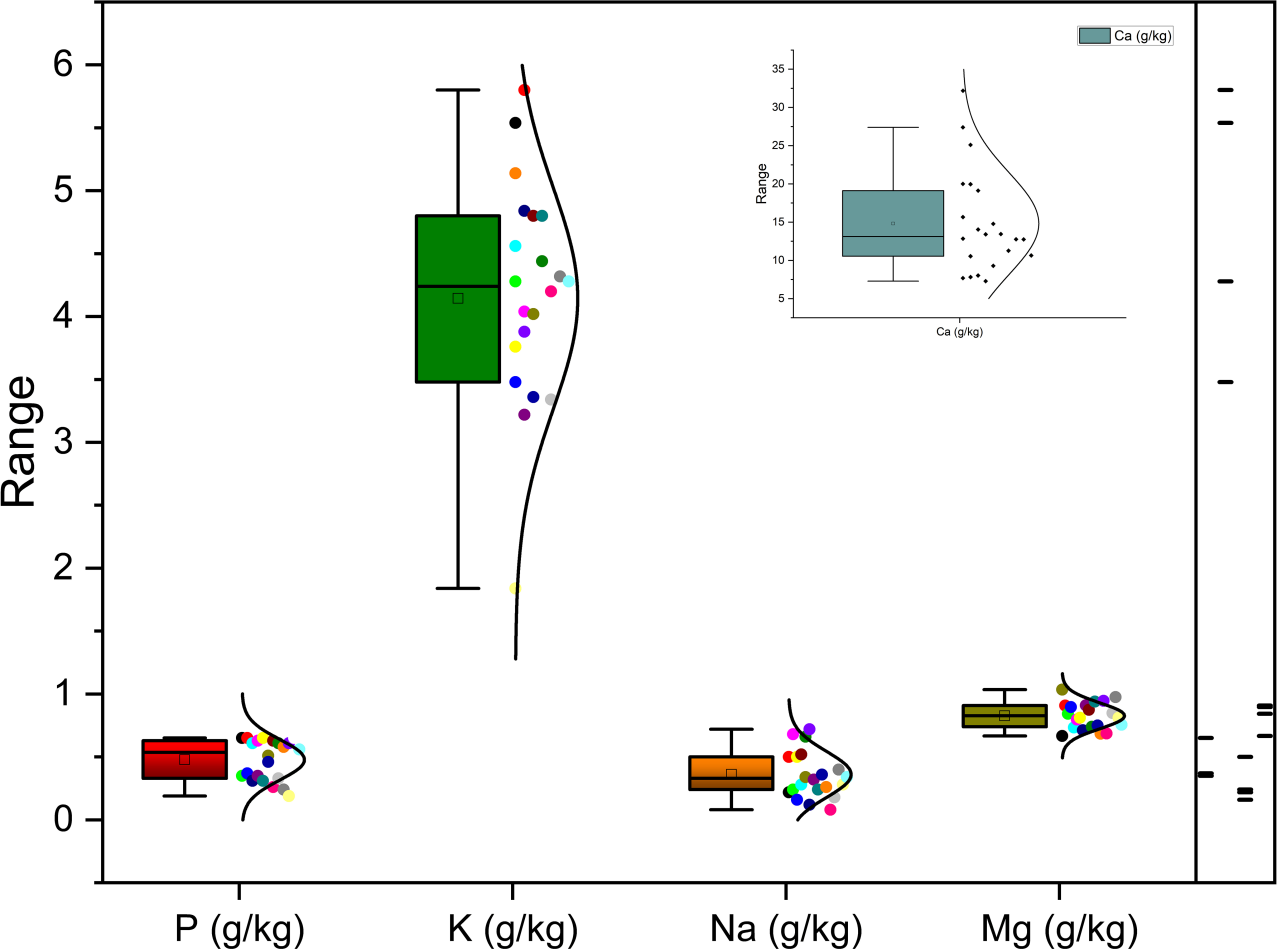
Diversity in macro-elements (P, K, NA, Ca and Mg) across investigated fig seed cultivars.

Richness of fig seeds in proteins and mineral elements has already been reported (Duman et al., 2018; Nakilcioğlu-Tas, 2019; Bölek, 2020); also, the effect of pollination on their content (Hssaini et al., 2022). Ustun-Argon et al. (2021) report that Potassium, Magnesium and Calcium were the main mineral compounds in the dried fig seeds. Our investigation showed similar results. Nevertheless, identification of genetic material that combines both agronomic performance and good nutritional quality is a major objective of genetic improvement programs for agricultural organisms and nurseries. During the past ten years, biofortification has gained prominence as a way of enhancing the nutritional profile of food crops (Jha and Warkentin, 2020). This is how this investigation was initiated, which remains to the best of our knowledge the first to highlight the genotypically effect of fig seeds diversity on the protein composition and mineral elements. A potential effect of biotic and abiotic factors, as well as essential chemical inputs, on fruit and seed cultivation and quality exists. To this end, genotypes were methodologically cultured under similar experimental conditions to circumvent environmental variability, and the discrimination collected is potentially linked to the genetic factor of the matrix. Thus, analysis of results presented here, first, joined the previous investigations on the presence in figs seeds ten mineral elements including Nitrogen (N), Phosphorus (P), Potassium (K), Calcium (Ca), Sodium (Na), Iron (Iron), Zinc (Zn), Manganese (Mn), Copper (Cu) and Magnesium (Mg), which showed as expected statistically significant (P<0.05) and non-significant differences between genotypes. Among macro-elements (P, K, Na, Ca and Mg) analyzed, calcium and potassium were the most abundant elements in the figs seeds genotypes studied (**Figure 1**). As the visual representation alone did not provide a comprehensive understanding of the phenotypic diversity, we recognized the need to supplement it with numerical data. In order to achieve a more thorough analysis, we included **Tables 3A**, **B** which presents the experimental data for the nutritional elements found in fig seeds, combined with the ANOVA results for fig samples mineral composition. This additional data not only supports our findings, but also offers a more detailed perspective on the observed phenotypic diversity.

**Table 3A:** Statistical analysis of the nutritional elements of fig (*Ficus carica* L.) seeds.

Fig seed samples	NT (%)	Proteins (%)	P (g/kg)	K (g/kg)	Ca (g/kg)	Na (g/kg)	Mg (g/kg)	Fe (mg/kg)	Zn (mg/kg)	Mn (mg/kg)	Cu (mg/kg)
Bourqui 2210	3.56 ± 0.06	22.27 ± 0.3	0.65 ± 0.05	5.54 ± 0.02	12.84 ± 0.04	0.22 ± 0.10	0.667 ± 0.00	94.5 ± 3.85	20.45 ± 0.80	15.48 ± 0.08	27.43 ± 0.48
Melissosyki 3074	2.83 ± 0.14	17.68 ± 0.88	0.65 ± 0.00	5.8 ± 0.28	15.66 ± 0.02	0.5 ± 0.02	0.909 ± 2.50	145.15 ± 1.00	36.95 ± 4.80	16.28 ± 0.33	24 ± 0.05
Embar El Khal 2247	2 ± 0.04	12.51 ± 0.26	0.35 ± 0.02	4.28 ± 0.40	10.54 ± 0.46	0.24 ± 0.04	0.842 ± 1.40	98.23 ± 4.27	26.13 ± 3.33	17.65 ± 0.60	31.28 ± 0.03
Kamalata 3075	2.74 ± 0.02	17.11 ± 0.13	0.37 ± 0.00	3.48 ± 0.00	14.04 ± 2.20	0.16 ± 0.00	0.896 ± 25.00	230.78 ± 10.18	20.18 ± 4.03	17.48 ± 1.68	34.73 ± 1.43
Adroulaniki 3073	2.55 ± 0.01	15.93 ± 0.09	0.61 ± 0.00	4.56 ± 0.04	13.4 ± 0.20	0.28 ± 0.08	0.734 ± 8.00	105.13 ± 0.68	16.35 ± 2.25	17.9 ± 0.45	23.53 ± 0.12
Amtalaa Arch 2210	3.29 ± 0.15	20.56 ± 0.96	0.63 ± 0.02	4.04 ± 0.00	7.68 ± 0.32	0.68 ± 0.24	0.797 ± 0.00	120.13 ± 3.43	37.53 ± 0.38	19.88 ± 0.28	27.18 ± 0.53
Lerida 2280	3.21 ± 0.04	20.04 ± 0.26	0.65 ± 0.00	3.76 ± 0.12	7.82 ± 0.70	0.5 ± 0.02	0.812 ± 13.75	108.98 ± 6.02	19.18 ± 1.93	16.23 ± 0.07	27.5 ± 1.10
Breba Blanca	2.39 ± 0.04	14.92 ± 0.22	0.51 ± 0.00	4.02 ± 0.14	20 ± 0.12	0.34 ± 0.10	1.036 ± 4.25	94.3 ± 2.90	125.9 ± 41.40	10.13 ± 0.42	19.83 ± 0.28
Hayoul 2265	2.68 ± 0.01	16.76 ± 0.04	0.31 ± 0.02	4.84 ± 0.20	27.4 ± 1.04	0.12 ± 0.04	0.713 ± 1.75	281.25 ± 13.40	33.78 ± 2.68	12.83 ± 0.13	34.28 ± 0.42
INRA 2802	1.46 ± 0.01	9.1 ± 0.09	0.35 ± 0.02	3.22 ± 0.14	19.96 ± 0.52	0.32 ± 0.00	0.91 ± 18.75	51.73 ± 4.08	18.7 ± 1.05	8.28 ± 1.13	19.33 ± 0.88
Bourjassate Noire	3.12 ± 0.06	19.51 ± 0.35	0.63 ± 0.02	4.8 ± 0.16	14.78 ± 0.22	0.52 ± 0.32	0.875 ± 11.75	106.5 ± 10.05	29.58 ± 4.08	15.25 ± 1.10	25.83 ± 0.78
Breval Blanca 2736	2.86 ± 0.05	17.89 ± 0.31	0.61 ± 0.05	4.44 ± 0.12	8.02 ± 0.66	0.66 ± 0.18	0.74 ± 2.75	165.68 ± 0.78	16.08 ± 0.53	16.05 ± 0.25	30.23 ± 0.13
Aicha Moussa 2208	2.7 ± 0.01	16.89 ± 0.09	0.31 ± 0.02	4.8 ± 0.16	32.18 ± 0.50	0.24 ± 0.08	0.94 ± 9.00	332.58 ± 16.88	25.58 ± 1.38	13.05 ± 0.15	33.55 ± 0.30
Nabout 2893	2.07 ± 0.01	12.91 ± 0.04	0.46 ± 0.00	3.36 ± 0.00	7.28 ± 0.08	0.36 ± 0.04	0.75 ± 6.75	120.5 ± 10.70	20.38 ± 2.23	9.95 ± 0.50	23.93 ± 0.13
Grosse Dame Blanche 2953	2.85 ± 0.04	17.81 ± 0.22	0.58 ± 0.02	5.14 ± 0.10	13.44 ± 0.20	0.26 ± 0.02	0.685 ± 0.50	83.45 ± 3.05	28.98 ± 1.28	15.33 ± 0.28	25.2 ± 0.70
EL Qoti Lezreq 2883	2.87 ± 0.06	17.94 ± 0.35	0.61 ± 0.00	3.88 ± 0.00	9.28 ± 0.16	0.72 ± 0.04	0.945 ± 3.00	91.38 ± 0.93	32.88 ± 9.93	14.7 ± 0.10	24.38 ± 0.07
Hafer Jmel 2253	2.28 ± 0.03	14.22 ± 0.22	0.26 ± 0.02	4.2 ± 0.08	25.1 ± 0.82	0.08 ± 0.04	0.686 ± 0.00	87.93 ± 24.93	11.93 ± 2.88	15.33 ± 0.33	25.48 ± 0.73
Rhoult 2216	3.86 ± 0.37	24.11 ± 2.32	0.65 ± 0.05	3.24 ± 0.08	11.26 ± 0.54	0.54 ± 0.06	0.867 ± 2.75	155.88 ± 8.83	28.53 ± 1.08	20.08 ± 0.23	25.68 ± 0.63
White Adriatic 102	2.09 ± 0.04	13.08 ± 0.22	0.33 ± 0.00	3.34 ± 0.06	12.76 ± 0.24	0.18 ± 0.02	0.848 ± 63.25	72.13 ± 23.58	12.23 ± 6.93	17.33 ± 1.58	27.55 ± 0.45
INRA 2603	2.13 ± 0.06	13.3 ± 0.35	0.24 ± 0.00	4.32 ± 0.04	12.74 ± 0.74	0.4 ± 0.12	0.975 ± 18.50	115.1 ± 0.45	25.4 ± 1.10	23.58 ± 0.57	28.4 ± 0.60
INRA 2105	0.97 ± 0.01	6.08 ± 0.04	0.19 ± 0.00	1.84 ± 0.00	19.12 ± 0.52	0.28 ± 0.04	0.811 ± 96.00	38.98 ± 6.33	4.55 ± 2.35	6.03 ± 1.33	23.45 ± 1.55
Ahra 2870	3.22 ± 0.00	20.13 ± 0.00	0.56 ± 0.00	4.28 ± 0.00	10.64 ± 0.24	0.34 ± 0.10	0.755 ± 2.25	117.9 ± 2.50	26.55 ± 1.90	9.38 ± 0.82	18.38 ± 0.32

NT, Total Nitrogen; P, Phosphorus; K, Potassium; Ca, Calcium; Na, Sodium; Mg, Magnesium; Fe, Iron; Zn, Zinc; Mn, Manganese; Cu, Copper.

**Table 3B:** Results of the Analysis of Variance (ANOVA).

	Sum of Squares	df	Mean Square	F	Sig.
NT (%)	30.230	25	1.209	134.519	0.000
Proteins (%)	1180.863	25	47.235	134.519	0.000
P (g/kg)	1.941	25	0.078	160.060	0.000
K (g/kg)	70.165	25	2.807	157.779	0.000
Ca (g/kg)	3174.203	25	126.968	285.853	0.000
Na (g/kg)	3.297	25	0.132	11.020	0.000
Mg (g/kg)	777727.635	25	31109.105	50.343	0.000
Fe (mg/kg)	368579.086	25	14743.163	117.277	0.000
Zn (mg/kg)	35424.697	25	1416.988	16.802	0.000
Mn (mg/kg)	1570.680	25	62.827	66.204	0.000
Cu (mg/kg)	1292.888	25	51.716	71.766	0.000

ANOVA, Analyze of variance; Sig., Significance of ANOVA.

**Figure 2:**
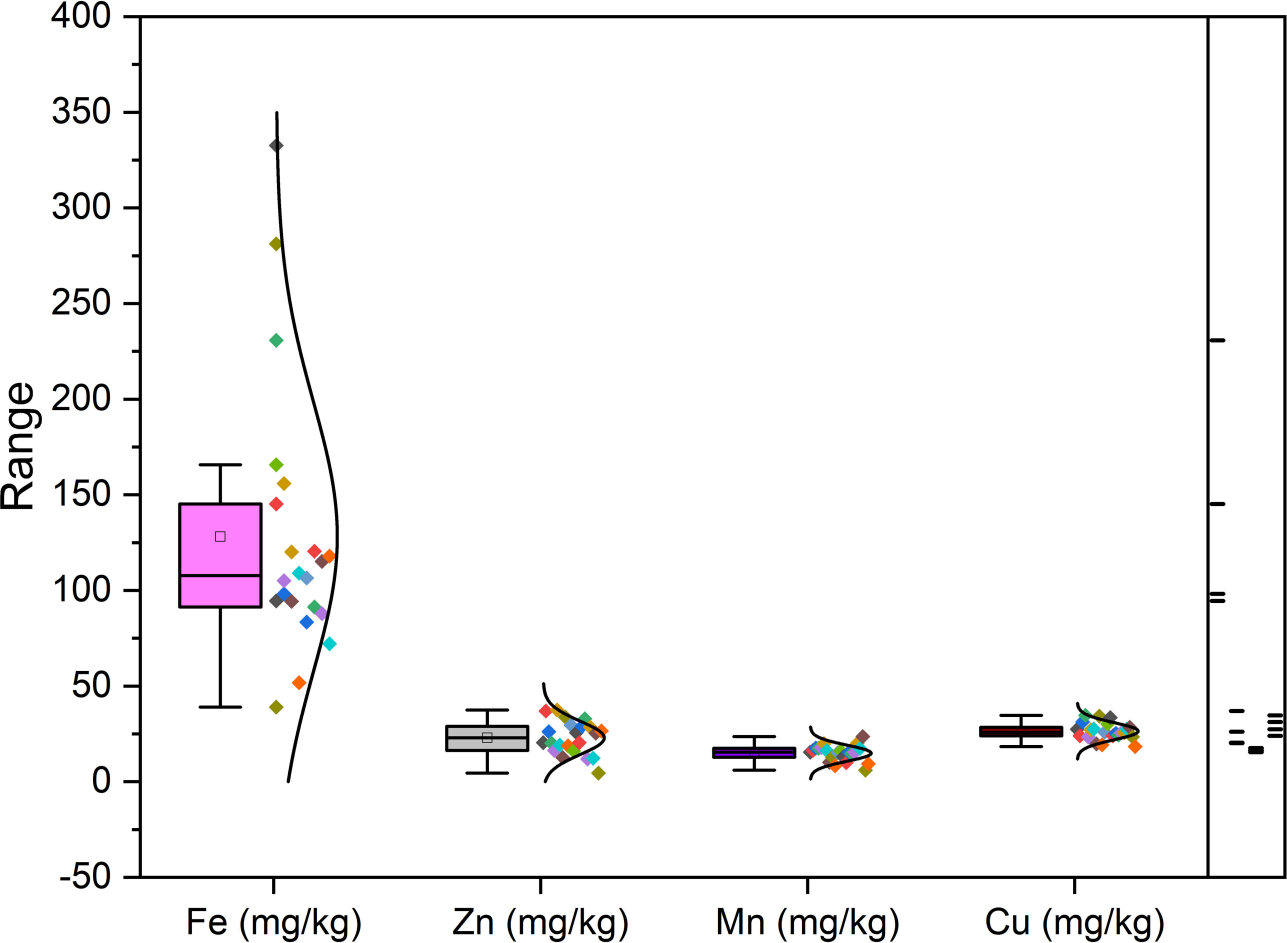
Variability of micro-elements (Fe, Zn, Mn and Cu) in fig seed genotypes.

According to the data presented in **Table 3A**, it’s evident that the Ca content in the different fig seed genotypes varied significantly. The highest concentration of Ca was observed in the “Aicha Moussa” genotype, with a value of 32.18 ± 0.50 (g/kg), whereas the lowest concentration was found in the “Nabout” genotype, with a value of 7.28 ± 0.08 (g/kg). Similarly, Potassium was present in significant amounts in all the sampled genotypes, with values ranging from 1.84 ± 0.00 (g/kg) in the “INRA 2105” genotype to 5.8 ± 0.28 (g/kg) in the “Melissosyki” genotype. In addition to the macro-elements, the results from the study also revealed that the sampled genotypes had a substantial content of Iron compared to other micro-elements (as depicted in **Figure 2**). The Iron content ranged from a minimum of 38.98 ± 6.33 mg/kg observed in the “INRA 2105” genotype to a maximum of 332.58 ± 16.88 mg/kg recorded in the “Aicha Moussa” genotype.

**Figure 3:**
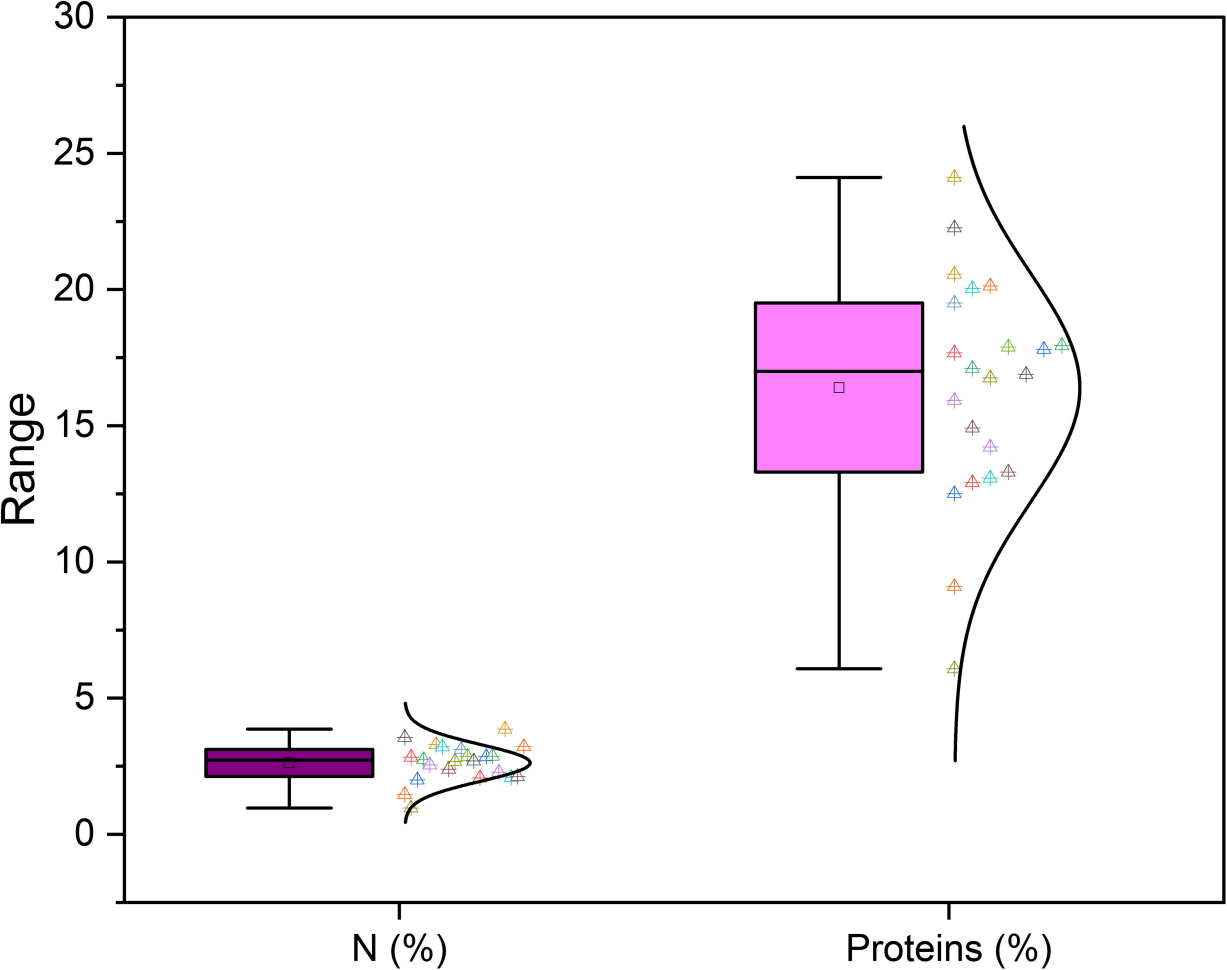
Variation in nitrogen and protein content among studied fig seed cultivars.

Furthermore, Nakilcioğlu-Tas (2019) reported the protein content of fig seeds between 14.74-15.07%, and Ustun-Argon et al. (2021) found 14.65%. Whereas, in our investigation, protein content varied between a minimum of 6.08 ± 0.04% recorded in the “INRA 2105” genotype and a maximum of 24.11 ± 2.32% recorded in the “Rhoul” genotype. As a comparison with the content of other fruit seeds, protein content of mangosteen seeds is 6.57%, 7.6% for guava, 12.4% for rambutan, 25.0% for honeydew, 25.63% for papaya, 25.2 to 37% for watermelon and 39.25% for pumpkin (Raihana et al., 2015). Moreover, between nitrogen and proteins, proteins are logically the most abundant in the fig seed genotypes studied (**Figure 3**). In agreement with the finding of Hssaini et al. (2022), it seems that minerals and proteins content of fig seeds depends on pollination and then on the source of pollen, which requires further studies able to investigate the qualitative performance of female genotypes sampled by compared to crossing with any caprifig tree. Since fig seeds have not received particular attention until now compared to the other parts of the species, results reported here constitute a solid base which will allow the orientation of new investigations.

### 3.3 Pairwise correlations between nutritional elements on fig seeds genotypes

**Figure 4:**
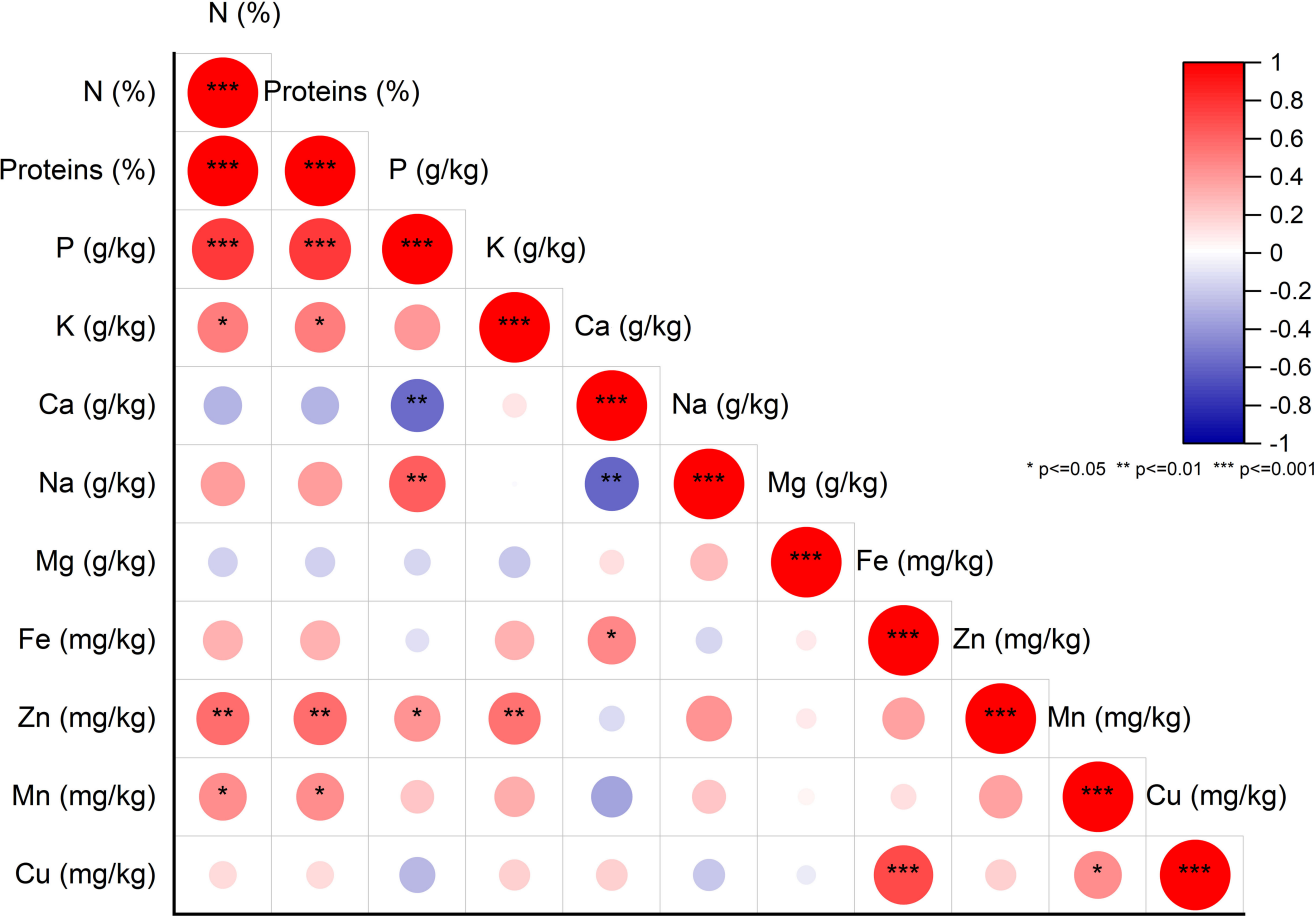
Pearson Pairwise correlation analysis of nutritional element content. Positive correlations (+) displayed in red and negative correlations (-) in blue. Color intensity corresponds to correlation coefficient magnitude. Color legend depicts correlation coefficients and respective colors.

To explore potential connections between protein content and mineral levels in fig seeds, we conducted a pairwise correlation analysis using the Pearson coefficient and presented the results in **Figure 4**. Weak correlations between the variables are represented by a low color intensity, while strong correlations are indicated by a high color intensity. Only correlations that were statistically significant at the 0.001 level with correlation coefficients greater than or equal to |0.5| were considered noteworthy. The matrix heatmap generated from the correlation analysis revealed a highly significant positive correlation between protein content and phosphorus content. We also observed a significant positive correlation between copper and iron levels. Additionally, bivariate correlations of the variables showed positive weakly correlated associations between manganese and protein as well as potassium. However, a highly significant negative correlation was detected between the calcium content and both phosphorus and sodium levels. Interestingly, our results differ from those of previous studies that found a significant positive correlation between iron and zinc concentrations in lentil seeds (Choukri et al., 2020). No such correlation was observed in the analyzed fig seed genotypes. Our findings align with those of other researchers, such as Kumar et al. (2014) and Darai et al. (2020), who reported similar observations of no correlation between iron and zinc levels. These results hold significant implications for future fig seed breeding programs, as they can serve as biomarkers to predict the level of significance, strength, and direction of associations between different variables. Examining potential correlations in the data set provides valuable insights into the most critical factors for evaluating and classifying fig seed genotypes.

### 3.4 Two-dimensional clustered heatmap analysis

**Figure 5:**
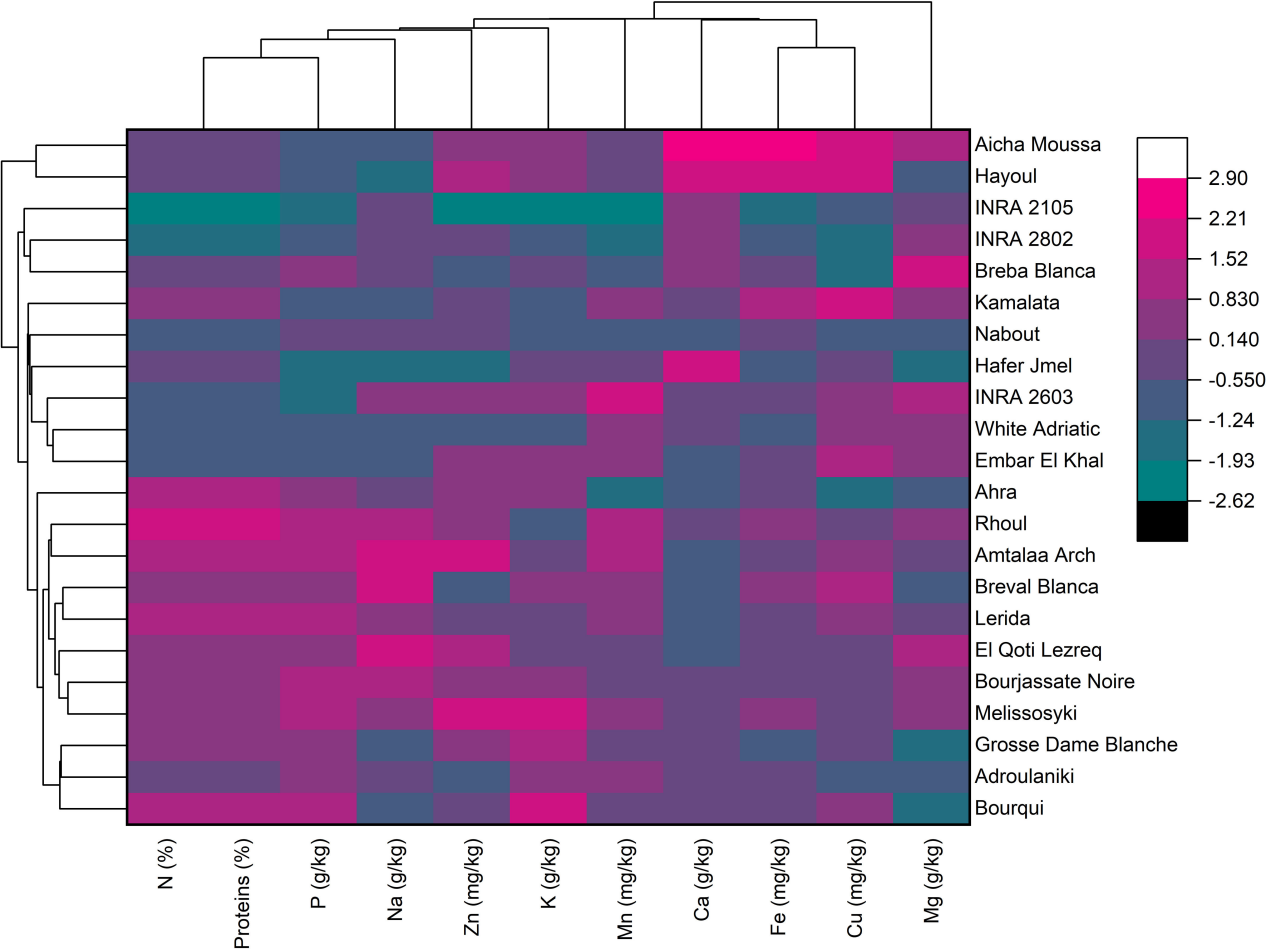
Two-dimensional hierarchical clustering heatmap based on the correlation matrix of nutrient elements and proteins in fig seed genotypes.

The hierarchically clustered heatmap is a useful tool for analyzing complex biological data without reducing the dimensionality. This technique displays network connections in a symmetric adjacency matrix, providing a two-dimensional color-coded heatmap (Clark and Ma’ayan, 2011). The heatmap was formed with two groupings using the Euclidean distance and the Ward’s method. The first grouping was oriented for fig seed genotypes, while the second was for mineral elements and proteins, with a color indicating numerical differences in the correlation coefficient (**Figure 5**). The heatmap showed that Calcium and Iron had the highest scores in the data set, meaning they had the greatest impact on genotype clustering. However, other variables also showed an impact, although they were less important than these two elements. The combined dendrogram in **Figure 5** differentiated the fig seed genotypes into four main clusters. The first cluster included two genotypes; “Hayoul” and “Aicha Moussa”, which were particularly very rich in Ca, Fe and Cu, and moderately rich in Zn, K, proteins and poor in P and Na. The second cluster included nine genotypes composed of “INRA 2105”, “INRA 2802”, “Breba Blanca”, “Kamalata”, “Nabout”, “Hafer Jmel”, “INRA 2603”, “White Adriatic” and “Embar El Khal”, which were rich in Mg, Cu and poor in proteins, P and Na. The third cluster consisted of genotypes including “Ahra”, “Rhoul”, “Amtalaa Arch”, “Breval Blanca”, “Lerida”, “El Qoti Lezreq”, “Bourjassate Noire” and “Melissosyki”, which were grouped based on their poverty in Ca compared to other clusters but were rich in proteins, P, Na, and Zn compared to other clusters. The last cluster included the genotypes “Grosse Dame Blanche”, “Adroulaniki” and “Bourqui”, which were mainly grouped by their richness in proteins, P, K and moderate values of Zn, Mn, Ca, and Fe but poor in Na and Mg. Finally, the heatmap combined to PCA (Principal Component Analysis) below allowed a better understanding of the relationship between the variables and the resulting clusters. The heatmap was a supervised variable construction method, while the PCA was an unsupervised method.

### 3.5 PCA of minerals and protein content

**Figure 6:**
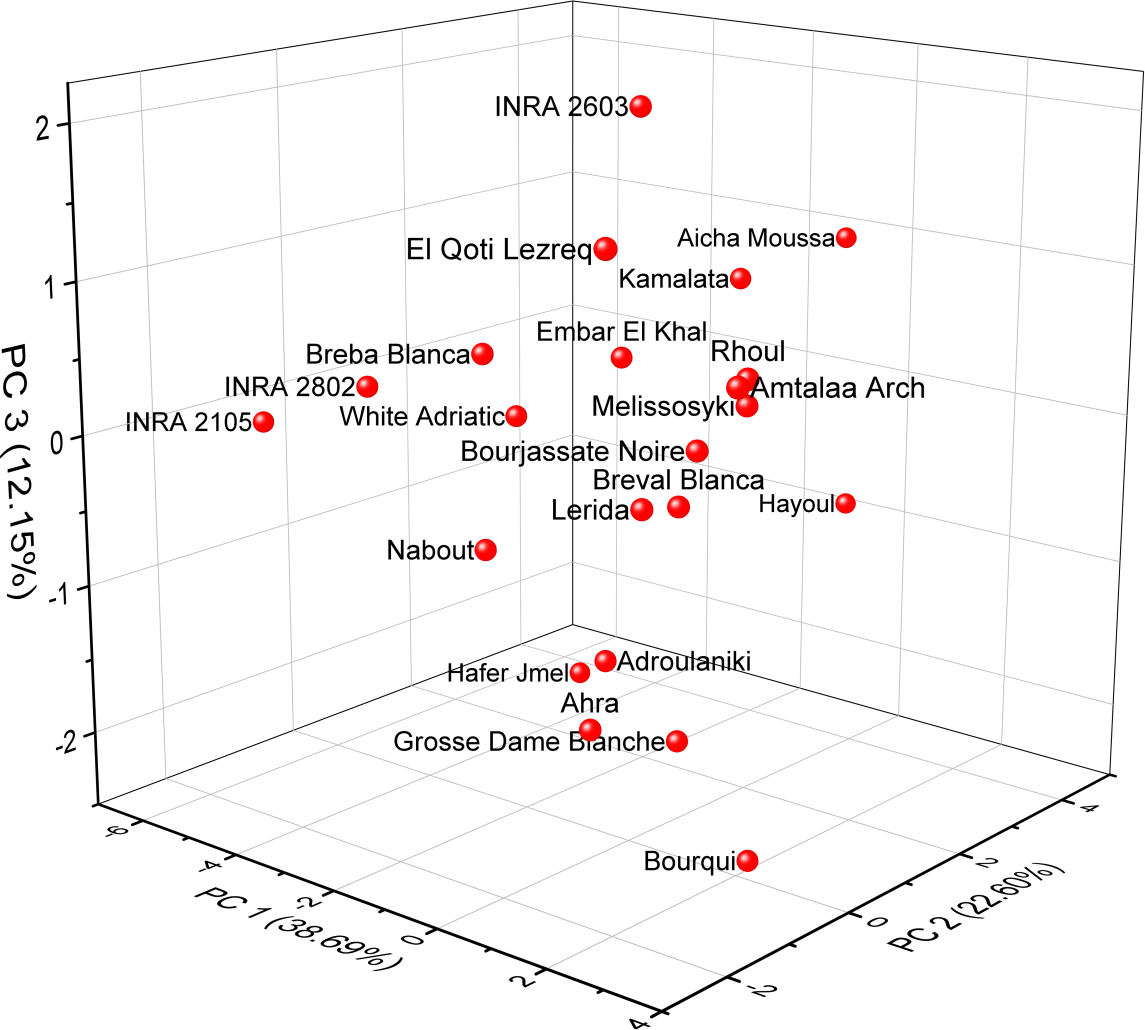
Principal component analysis (PCA) score plots derived from three components of mineral and protein content dataset.

Based on the correlation coefficient, a principal component analysis (PCA) was performed to determine the discriminating variables in the data set. This analysis aimed to define the main factors that contribute to the discrimination of fig seeds. In our study, only a principal component loading of more than |0.35| was considered as being significant for each factor. The analysis of nutritional data using PCA revealed valuable insights into the minerals and protein content of fig seed cultivars. The results showed that three principal components were enough to explain 73.44% of the total variance, with the first component accounting for the majority at 38.69% (**Figure 6**). With reference to **Table 4**, the first component (PC1) consisted of the variables, N, P and Zn, for which the scores were respectively 0.45075, 0.39814 and 0.35065. It explained about 38.69% of the total variance observed, which means that these attributes had the highest impact on the discrimination. The second component (PC2) accounted for about 22.60% of the total variance and was defined by Fe, Cu and Ca, for which the respective scores were 0.53085, 0.49685 and 0.43542. The third component (PC3) represented 12.15% of the total inertia and was mainly correlated with the amount of Mg (0.7528) and Na (0.39577). This information reported here could be used to inform on the distribution of these compositions between all genotypes studied, and could serve as a starting point for further research into the relationships between fig seed phenotypic attributes and nutritional properties.

**Table 4:** Eigenvectors of principal component axes from PCA analysis.

	Coefficients of PC1	Coefficients of PC2	Coefficients of PC3
N (%)	0.45075	0.04115	-0.12927
Proteins (%)	0.45079	0.04168	-0.12954
P (g/kg)	0.39814	-0.27596	-0.14759
K (g/kg)	0.29419	0.23866	-0.25989
Ca (g/kg)	-0.21076	0.43542	-0.05608
Na (g/kg)	0.27295	-0.34175	0.39577
Mg (g/kg)	-0.05464	-0.02922	0.7528
Fe (mg/kg)	0.12815	0.53085	0.15065
Zn (mg/kg)	0.35065	0.12292	0.19788
Mn (mg/kg)	0.28236	0.11153	0.24865
Cu (mg/kg)	0.08685	0.49685	0.16435

### 3.6 Characteristics of FTIR-ATR signatures

**Figure 7:**
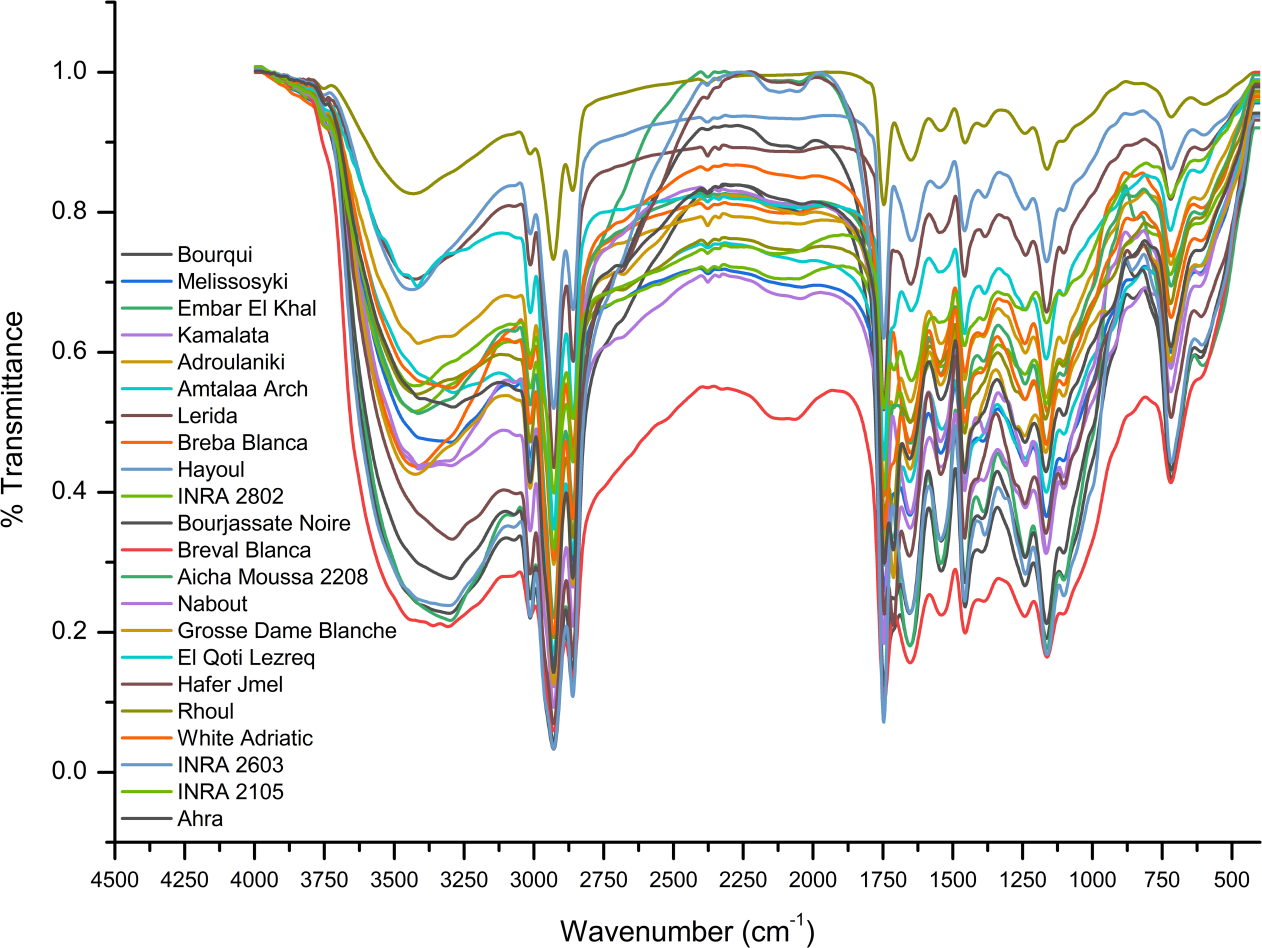
FTIR-ATR spectroscopy data of fig seeds in the wavenumber range from 4000 to 450 cm^-1^.

**Figure 8:**
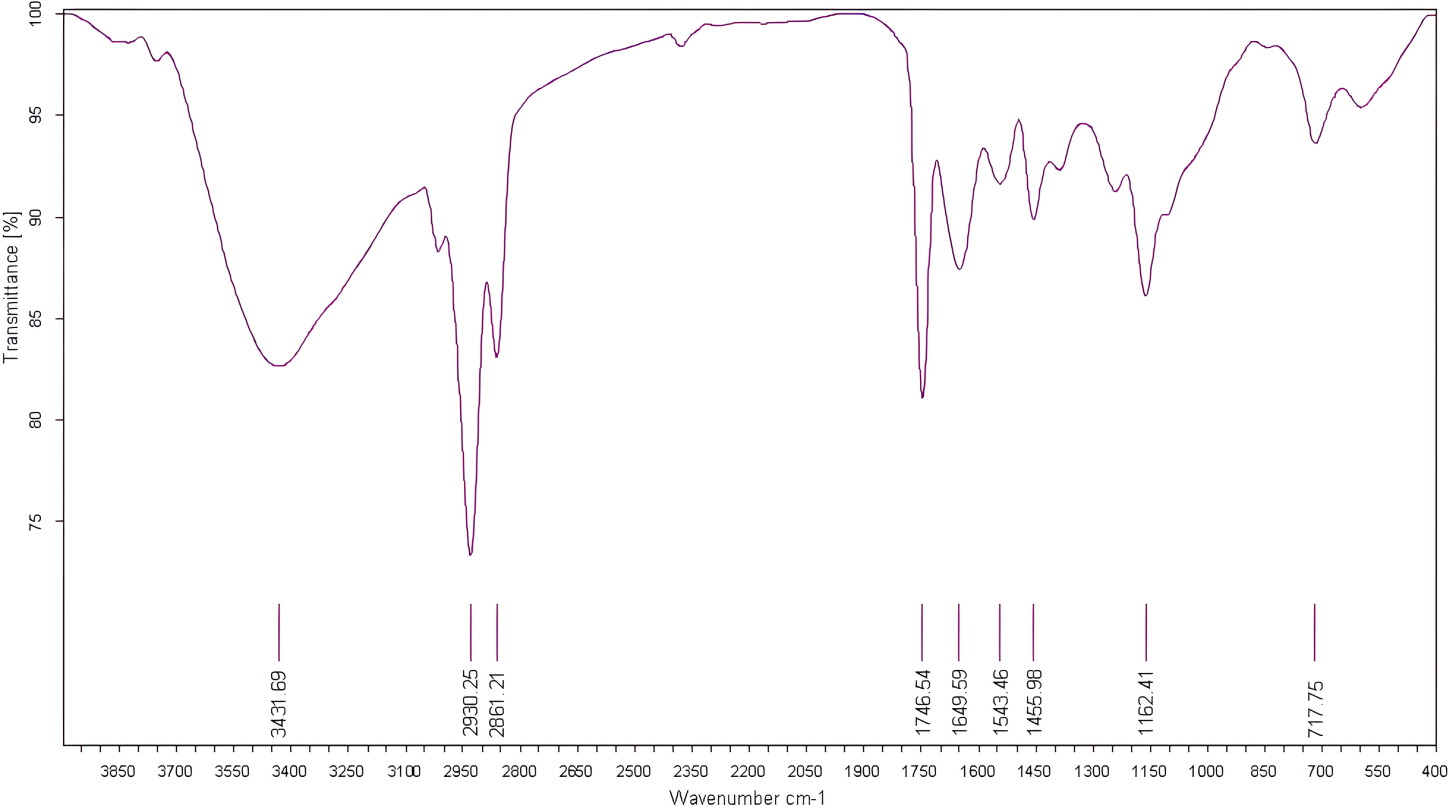
FTIR spectra of the local clone 'El Qoti Lezreq' fig seeds highlighting key peaks in the wavenumber range of 4000 to 450 cm^-1^.

For evaluation of fig seeds chemotypic properties, FTIR-ATR fingerprinting has proven to be a powerful and interesting approach for screening and discriminating the characteristic signatures present in the analyzed samples (Hssaini et al., 2021a). Also, this technique has provided in previous studies satisfactory results with a high-throughput screening framework providing numerous biomolecules in several food samples (De la Mata et al., 2012; Anjos et al., 2015; Cassani et al., 2017). In this sense, FTIR spectroscopy coupled with an ATR was used in this work to assess relevance of chemotypic discrimination, and to identify the characteristic molecular signatures of biochemical substances present in fig seeds in order to study their sensitivity to phenotypic diversity. Therefore, seeds samples were directly scanned in the wavenumber range of 4000 to 450 cm^-1^ with a spectral resolution of 4 cm^-1^. **Figure 7** illustrates spectra of the 22 fig seeds cultivars. Obviously, as expected for all seeds, all the spectra are similar. However, using chemometric tools, analysis of these spectra reveals important differences, particularly by evaluating their integrated intensities for each major vibrational region. These differences, even if sometimes considered minor, indicate molecular dissimilarities between samples. Overall, the peaks of each spectrum were identified at different wavenumbers, assigned to specific functional groups (Koch et al., 2014) and summarized in **Table 5**. The latter reports the detailed attributions of the bands, which reveal fifteen identifiable footprints in **Figure 8**. Indeed, **Figure 8** represents a unitary case for the genotype “El Qoti Lezreq”, which perfectly shows values of the wavelengths from 4000 to 450 cm^-1^ of the large vibratory regions with their corresponding peaks.

**Table 5:** FTIR peaks assignments for functional groups found in fig (*Ficus carica* L.) seeds spectrum.

Wavenumber (cm^-1^)	Functional groups	Modes of vibration	Assignments	References
3417.51	δ(O–H)	Stretching vibration	Intramolecular hydrogen bond between C(3)OH• • •O(5) and C(6)O• • •O(2)H (fibers)	Schwanninger et al. (2004); Oh et al. (2005) and Cassani et al. (2017)
3071.26	ν(=C–H)	Bending vibration	Unsaturated fatty acids	Oh et al. (2005); Bouafif et al. (2008); Gopalakrishnan and Raghu (2014) and Niu et al. (2017)
3012.70	δ(C–H), δ(O–H), δ(N–H)	Stretching vibration	Carboxylic acids, carbohydrates and phenolic compounds	Oh et al. (2005) and Bouafif et al. (2008)
2929.11 2860.06	δ(C–H), δ(–CH2–), δ(–CH3)	Stretching vibration	Methylene and methyl groups (lipids)	Niu et al. (2017) and Tulukcu et al. (2019)
1746.56	δ(COH), δ(OCH)	Stretching vibration	Ester carbonyl functional group of the triglycerides and fatty acids	Ahmed et al. (2005); Oh et al. (2005); Vlachos et al. (2006); De la Mata et al. (2012) and Tulukcu et al. (2019)
1648.75	δ(COH), δ(CCH), δ(OCH), ν(C–O)	Stretching and bending vibration	β-Sheet of amide I	Ellepola et al. (2005) and Tulukcu et al. (2019)
1544.32	δ(N–H), ν(C–N)	Stretching and bending vibrations	Amide II (α-Helix)	Ellepola et al. (2005) and Tulukcu et al. (2019)
1458.15	δ(C–H), ν(C–H)	Stretching and bending vibrations	Lipids, protein and cholesterol esters	Ahmed et al. (2005); Vlachos et al. (2006) and Liu et al. (2021)
1386.02 1239.31	ν(C–H), δ(C–O),	Stretching and bending vibrations	Esters	Sylverstein et al. (1991); Guillén and Cabo (1997); Ahmed et al. (2005) and Niu et al. (2017)
1165.91	δ(C–O), ν(C–C)	Bending vibration	Esters	Ahmed et al. (2005) and Niu et al. (2017)
1102.25	δ(C–O)	Stretching vibration	Unsaturated esters and esters derived from secondary alcohols	Guillén and Cabo (1997)
721.19	ν(–HC=CH– (*cis*-) and (*trans*-)	Bending vibration out of plane	Aromatic hydrocarbons	Guillén and Cabo (1997); Gopalakrishnan and Raghu (2014)
611.93	ν(–(CH2)_n_–),δ(–HC=CH–(*cis*-))	Bending vibration (rocking)	Disubstituted olefinic *cis*-alkenes	Van de Voort et al. (1995); Vlachos et al. (2006)

The first FTIR fingerprint appeared around 3417.51 cm^-1^ which would be assigned to O–H stretching vibration, most likely attributed to fibers, which have been reported to be present in large quantities in fig seeds (Bölek, 2020). This flattened peak appeared in the vibrational region from 3750 to 3100 cm^-1^ associated with intramolecular hydrogen bonding in cellulose between C(3)OH•••O(5) and C(6)O•••O(2)H according to Schwanninger et al. (2004); Oh et al. (2005) and Cassani et al. (2017). A second vibration can be detected between 3250 and 3000 cm^-1^. This occurred around 3071.26 cm^-1^, which is most likely attributed to C–H stretching of olefinic double bonds bonded to long carbon chains containing a relatively high number of unsaturated fatty acid CH_2_ groups (Oh et al., 2005; Bouafif et al., 2008; Gopalakrishnan and Raghu, 2014; Niu et al., 2017). Absorbance in the vibrational region from 3100 to 2950 cm^-1^ has been attributed to symmetric and asymmetric stretching of C–H, O–H and NH_3_. The peak appears around 3012.70 cm^-1^, which is probably typical for carboxylic acids, carbohydrates and phenolic compounds (Oh et al., 2005; Bouafif et al., 2008). Peaks in the range of 2950 to 2800 cm^-1^ are identified as absorbances related to asymmetric and symmetric stretching of aliphatic C–H in the −CH_2_ and terminal −CH_3_ groups, respectively (Niu et al., 2017; Tulukcu et al., 2019). A first sharp and distinct peak is at 2929.11 cm^-1^ and a second occurred at 2860.06 cm^-1^. Bands in the range of 1775 to 1725 cm^-1^ are attributed to stretching of ester carbonyl groups of triglycerides (Tulukcu et al., 2019). This absorbance region is related to C=O elongation of carboxylic ester type (Ahmed et al., 2005; Vlachos et al., 2006). At 1746.56 cm^-1^, a sharp peak was identified, and is probably due to C=O stretching vibration of carbonyl groups belonging to triacylglycerols. It’s probably associated with lipids and fatty acids contained in seeds (Oh et al., 2005; De la Mata et al., 2012). Nevertheless, several authors report that this peak could be attributed to proteins (Cai and Singh, 2004; Cocchi et al., 2004; Cocchi et al., 2006; Terpugov et al., 2016).

Amide I and Amide II bonds usually resulting from protein-bound structures. Vibrations in the wavenumber range of 1700 to 1550 cm^-1^ shows two peaks appeared at 1648.75 cm^-1^ and 1544.32 cm^-1^ which have been assigned to the Amide I and Amide II bands respectively (Ellepola et al., 2005; Tulukcu et al., 2019). According to several studies, this band between 1500 and 1100 cm^-1^ are the result of several weak peaks wouldn’t be differentiated in the analyzed samples, which would correspond to mixed vibrations resulting from bending modes of groups >CH_2_ and –CH_3_ in proteins, fatty acids and phosphate bearing compounds (Tulukcu et al., 2019). The spectral band at 1458.15 cm^-1^ could probably be associated with CH_2_ bending and methylene deformation of lipids, proteins or cholesterol esters (Ahmed et al., 2005; Vlachos et al., 2006; Liu et al., 2021). Vibrations occurred around 1386.02 and 1239.31 cm^-1^ could probably be assigned to CH_2_ shearing and C–O stretching vibrations attributed to esters (Ahmed et al., 2005). Indeed, absorptions of certain bending vibrations of the methylene group occur between 1350 and 1150 cm^-1^ (Guillén and Cabo, 1997). On the other hand, C–O bond stretching vibrations of esters are composed of two coupled asymmetric vibrations C–C(=O)–O and O–C–C, whose the former being larger (Sylverstein et al., 1991); these bands occur in the region between 1300 and 1000 cm^-1^. The C–C(=O)–O band of saturated esters appears between 1240 and 1163 cm^-1^, and in unsaturated esters, the vibration is produced at lower frequencies (Guillén and Cabo, 1997). Thus, the band appearing around 1165.91 cm^-1^ can be assigned to bending vibration of C–O ester group (Ahmed et al., 2005; Niu et al., 2017). However, O–C–C band of esters derived from primary alcohols appears in the range between 1064 and 1031 cm^-1^, whereas for those derived from secondary alcohols, the band appears at approximately 1100 cm^-1^; what can probably correspond to vibration appeared around 1102.25 cm^-1^ (Guillén and Cabo, 1997). Vibrational region ranging from 850 to 700 cm^-1^ is marked by a sharp peak appearing at 721.19 cm^-1^ which would probably be assigned to C–H deformation in aromatic hydrocarbons (Guillén and Cabo, 1997; Gopalakrishnan and Raghu, 2014). The last vibrational region ranging from 700 to 400 cm^-1^ shows a peak around 611.93 cm^-1^ attributed to overlap of bending vibrations of methylene −CH_2_ and out-of-plane vibrations of C–H bonds of disubstituted olefinic cis-alkenes (Van de Voort et al., 1995; Vlachos et al., 2006). As expected, although the fig seeds spectra appear to be similar and generally overlap in **Figure 7**, they show differences in integrated intensity of their bands as well as in the exact frequency at which maximum absorbance is produced in each case; probably because of genotypically differences in nature and composition.

**Figure 9:**
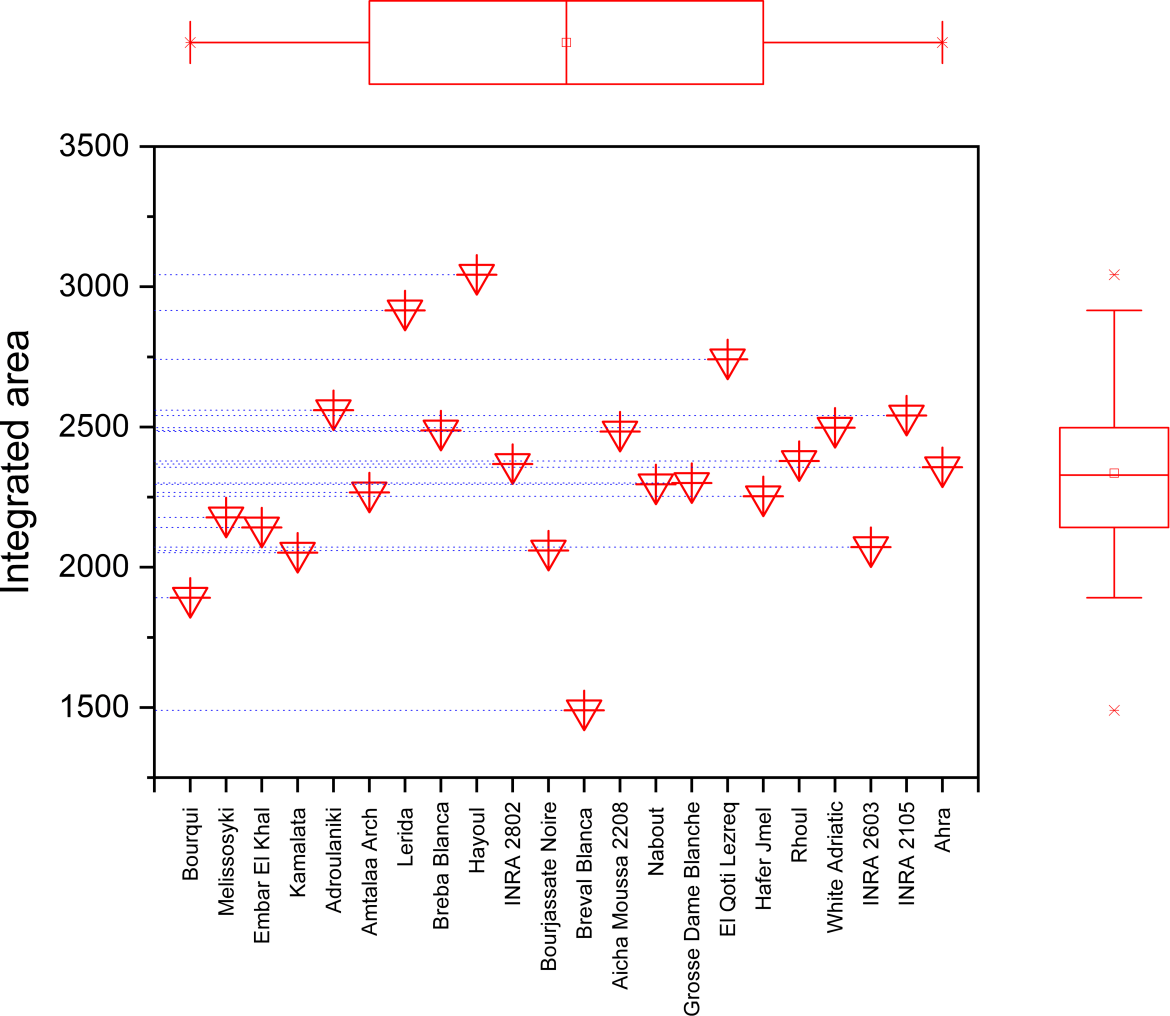
Marginal boxplot chart illustrating the total integrated area of the entire infrared (IR) spectrum for each fig seed cultivar.

The marginal boxplot chart in **Figure 9** provides a clear visualization of the phenotypic differences between the seed cultivars based on their vibrational intensities in the 3500-1000 cm^-1^ range. The chart displays the distribution of integrated intensities for each genotype, as well as the median, interquartile range, and outliers. The one-way analysis of variance conducted on the data revealed statistically significant differences in the integrated intensities of major peaks among the sampled fig seeds. For instance, the “Hafer Jmel” genotype had an integrated area of approximately 2250, while the “White Adriatic” genotype had an integrated area of around 2500. Interestingly, the “Breval Blanca” genotype had the lowest integrated area of about 1500, making it unique among the cultivars studied. These findings highlight the significant impact of genotype on the molecular signatures of fig seed cultivars. Although the differences may seem minor, they are crucial in determining the distribution of nutritional composition among the various genotypes. This is consistent with previous studies that have demonstrated a similar pattern of phenotypic effect on fig seed quality, including nutritional composition. Overall, the results suggest that FTIR-ATR spectroscopy could be an effective and non-destructive method for rapid analysis of the structural characteristics and functional properties of various genotypes of fig seeds. This could have important implications for improving breeding programs and enhancing the nutritional value of fig seeds for human consumption.

### 3.7 Multivariate analysis of FTIR-ATR spectra

**Figure 10:**
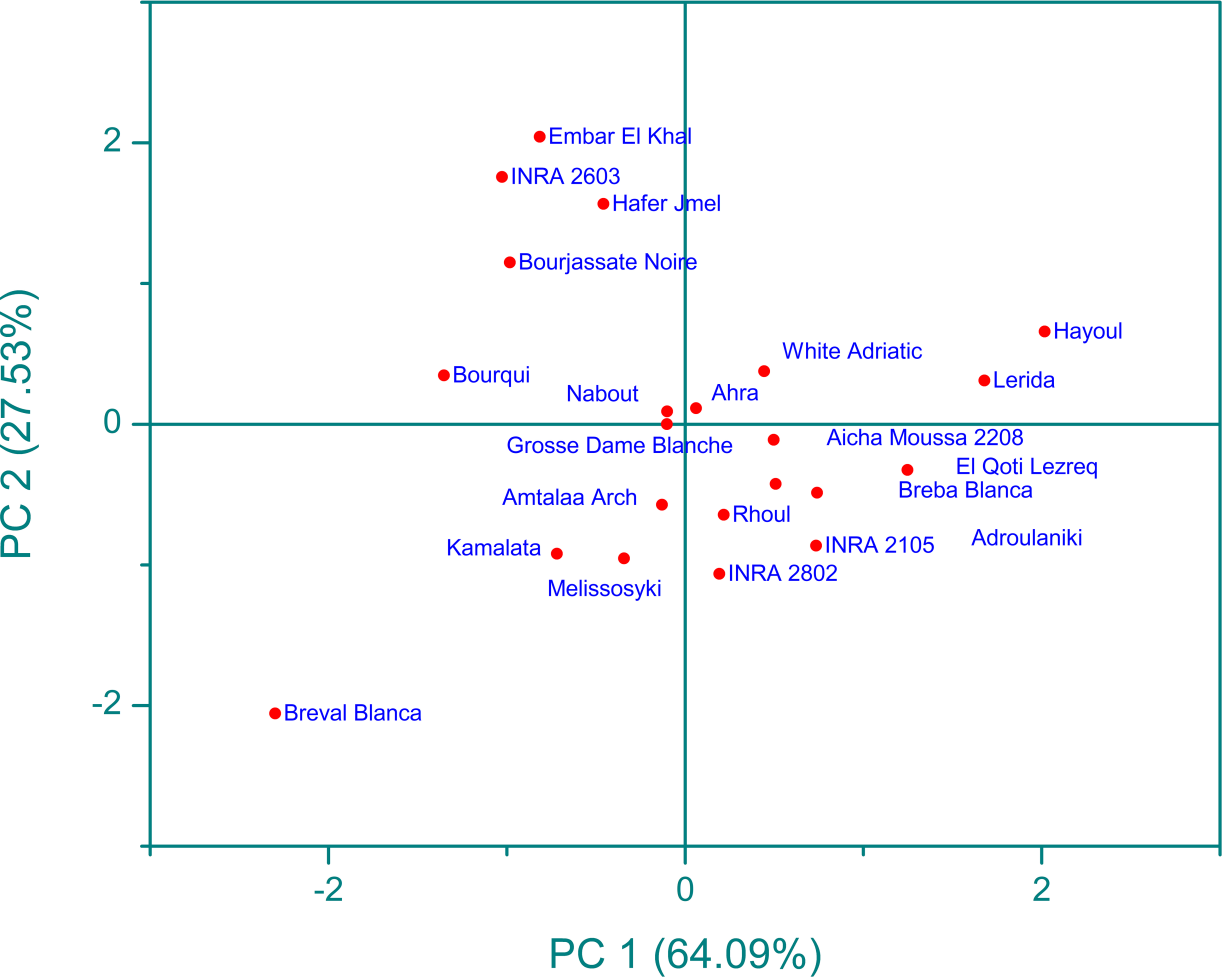
Principal component analysis (pca) score plot (pc1 x pc2) for ftir-atr spectra in the wavenumber range of 4000-450 cm^-1^.

Infrared spectroscopy or FTIR is a powerful analytical tool that has been widely used with chemometric methods such as PCA. By using PCA, the complexity of multi-wavelength FTIR data can be reduced to a limited number of parameters, with minimal loss of total variance. This process results in the creation of a scatterplot, which reveals the resolution of the classification of fig seed samples based on their phenotypic diversity. This unsupervised variable construction method was applied to spectra to unveil the resolution of fig seed classification based on phenotypic diversity. This technique was also employed to evaluate if spectra data could produce a discrimination pattern similar to that of nutritional composition. **Figure 10** presents FTIR data in the 4000-450 cm^-1^ wavenumber range, exhibiting a total variance of 91.62%. The first component (PC1) accounted for 64.09% of the variance, while the second component (PC2) contributed 27.53% to the total variance. The PCA plot displayed a high level of discrimination resolution, clustering cultivars with similar FTIR profiles, as spectra are a qualitative method that precisely measures functional groups.

The scatter plot of the PCA model reveals four main clusters, with clear classification of the “Breval Blanca” genotype, which showed the lowest vibration intensities among all cultivars studied. These four groups were differentiated based on the total intensity area in the entire vibration range. This result is in line with previous studies that used FTIR spectra and chemometric approaches to distinguish several fruit seeds, including apples and grapes. Previous studies using FTIR spectra coupled with chemometric approaches have demonstrated the excellent discrimination capacity of this method in differentiating various fruit seeds, such as apples (Anang et al., 2019) and grape (Lucarini et al., 2019). The results suggest that FTIR spectroscopy is a useful tool for the screening and discrimination of a large sample of fig seeds based on chemotypic attributes. It’s an accurate, rapid, inexpensive, and environmentally friendly technology that requires minimal sample preparation, making it a valuable tool for the management of fig seed diversity and for use in breeding and industrial development programs. This study is the first of its kind to assess the chemotypic attributes of a wide range of fig seed genotypes and has important implications for maintaining the longevity and genetic diversity of the species.

## 4 Conclusion

The findings of this study underscore the considerable potential of fig seeds as a rich source of lipids, proteins, and minerals. The results reveal substantial variations in the content of these essential nutrients among different fig genotypes, emphasizing the significance of exploring the phenotypic diversity of fig seeds. The two-dimensional clustered heatmap differentiated the fig seed genotypes into four distinct clusters based on their lipochemical profiles. Furthermore, the utilization of multivariate analysis for FTIR data proved effective in classifying the genotypes and providing insights into their chemotypic variability. This study contributes to the selection of potential fig seed cultivars for both scientific and industrial purposes, promoting the utilization of the chemotypic properties of fig seeds and unlocking their complete nutritional potential. The extracted oil from fig seeds holds promise as a food supplement or ingredient in various food products. However, to fully leverage the potential of fig seeds, further research is required, such as investigating techniques to increase oil yield and improve lipochemical properties by exploring environmental conditions like heat and salt stress. Additionally, assessing the bioavailability and bioaccessibility of nutrients present in fig seeds is crucial. Ultimately, this work represents a significant contribution to the selection of highly discriminant variables for optimizing the agronomic performance of fig seed genotypes. The findings also hold important implications for fig collection management, ensuring the preservation of species longevity and diversity, and facilitating its utilization in breeding programs.

## Data availability statement

The original contributions presented in the study are included in the article/supplementary material. Further inquiries can be directed to the corresponding author.

## Author contributions

LH conceived and performed the project; AI and LH conducted species identification and collected samples; LH designed and managed the experiments; LH, AI, RO, and RA performed the analyses and collected data; LH analyzed data and performed the chemometrics analysis; AI assisted with data analyses, interpreted and discussed the data and drafted the manuscript; RR and KH contributed to the data curation and visualization; LH revised the manuscript; All authors contributed to the article and approved the submitted version.
